# Introgression of mitochondrial DNA among *Myodes *voles: consequences for energetics?

**DOI:** 10.1186/1471-2148-11-355

**Published:** 2011-12-09

**Authors:** Zbyszek Boratyński, Paulo Célio Alves, Stefano Berto, Esa Koskela, Tapio Mappes, José Melo-Ferreira

**Affiliations:** 1 Centre of Excellence in Evolutionary Research, Department of Biological and Environmental Science, University of Jyväskylä, P.O. Box 35 YAC, Finland; 2 CIBIO, Centro de Investigação em Biodiversidade e Recursos Genéticos, Universidade do Porto, Campus Agrário de Vairão, 4485-661 Vairão, Portugal; 3 Departamento de Biologia, Faculdade de Ciências da Universidade do Porto, Rua do Campo Alegre, s/n, 4169-007 Porto, Portugal; 4 University of Montana, Wildlife Biology Program, College of Forestry and Conservation, Missoula, MT 59812, USA; 5 Department of Biological and Environmental Science, University of Jyväskylä, P.O. Box 35 YAC, Finland

## Abstract

**Background:**

Introgression of mitochondrial DNA (mtDNA) is among the most frequently described cases of reticulate evolution. The tendency of mtDNA to cross interspecific barriers is somewhat counter-intuitive considering the key function of enzymes that it encodes in the oxidative-phosphorylation process, which could give rise to hybrid dysfunction. How mtDNA reticulation affects the evolution of metabolic functions is, however, uncertain. Here we investigated how morpho-physiological traits vary in natural populations of a common rodent (the bank vole, *Myodes glareolus*) and whether this variation could be associated with mtDNA introgression. First, we confirmed that *M. glareolus *harbour mtDNA introgressed from *M. rutilus *by analyzing mtDNA (cytochrome b, 954 bp) and nuclear DNA (four markers; 2333 bp in total) sequence variation and reconstructing loci phylogenies among six natural populations in Finland. We then studied geographic variation in body size and basal metabolic rate (BMR) among the populations of *M. glareolus *and tested its relationship with mtDNA type.

**Results:**

*Myodes glareolus *and its arctic neighbour, *M. rutilus*, are reciprocally monophyletic at the analyzed nuclear DNA loci. In contrast, the two northernmost populations of *M. glareolus *have a fixed mitotype that is shared with *M. rutilus*, likely due to introgressive hybridization. The analyses of phenotypic traits revealed that the body mass and whole-body, but not mass corrected, BMR are significantly reduced in *M. glareolus *females from northern Finland that also have the introgressed mitotype. Restricting the analysis to the single population where the mitotypes coexist, the association of mtDNA type with whole-body BMR remained but those with mass corrected BMR and body mass did not. Mitochondrial sequence variation in the introgressed haplotypes is compatible with demographic growth of the populations, but may also be a result of positive selection.

**Conclusion:**

Our results show that the phenotypic traits vary markedly along the north-south axis of populations of *M. glareolus*. This variation may be related to adaptation to local environments and coincides with the gradient of genome reticulation between *M. glareolus *and *M. rutilus*, which was assessed by mtDNA introgression. Introgression of mtDNA may have affected morpho-physiological traits but do not show strong effects on either body mass or basal metabolic rate alone. We discuss the causes and biological meaning of our results and the means to clarify these questions in future research.

## Background

Widely distributed species face different selection pressures along climatic and ecological gradients. In this respect, basal metabolic rate (BMR) is particularly prone to evolve adaptively, since it has been shown to be heritable and correlates with fitness components in endotherms [1-8]. Several comparative studies have proposed that patterns of variation in metabolic rate match certain climatic variables in widely distributed groups of species [9,10], suggesting that the level of BMR responds to different selection regimes generated by local conditions. BMR is generated mainly in physiologically important internal organs including the kidneys, brain and liver [11,12] with most of the physiological activity localized in certain compartments of cells in these organs.

Mitochondria are the main cellular energy "factories", supplying organisms with energy stored in ATP molecules. The rate of energy production by mitochondria is crucial for an individual's fitness and is thought to be determined by natural selection [13-15]. Mitochondria are characterized by their own genome and harbour enzymatic elements of aerobic metabolic pathways. However, oxidative phosphorylation (OXPHOS), the process responsible for energy production, depends not only on the enzymes encoded in the internal organelle genome (mitochondrial DNA [mtDNA]), but also on nuclear-encoded polypeptides which interact to enable the enzymatic reactions. Therefore, selection may act on and constrain the co-evolution of mitochondrial and nuclear genes [16]. Interestingly, between-species transfer of mtDNA has been frequently observed among many group of animals and plants [17-22]. Such transfer has been traditionally considered neutral, however, it may be maladaptive, if introgression breaks the coevolved mito-nuclear complexes [16], or adaptive, as mtDNA function may represent important selective value [23-26].

**Figure 1:**
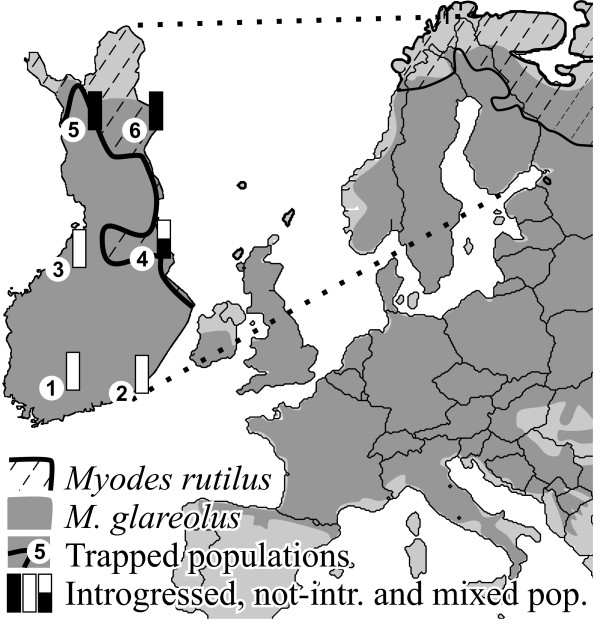
**Distribution of bank vole *Myodes glareolus *(gray) and red vole *M. rutilus *(striped area)**. Numbers in white circles on the enlarged map of Finland refer to the localizations of trapped populations: 1 - Tammela (SW), 2 - Virolahti (SE), 3 - Kannus (CW), 4 - Sotkamo (CE), 5 - Kolari (NW) and 6 - Savukoski (NE). Species distributions after: Amori et al. 2009 and Linzey et al. 2009.

Interspecific mtDNA transfer observed among the broadly distributed rodent genus *Myodes *[27-30] offers an ideal situation to test 1) if reticulate evolution has an important and recognizable phenotypic effect and 2) whether natural selection may influence mtDNA introgression. The bank vole, *Myodes glareolus*, is dispersed across different climatic and geographic zones, from the Mediterranean to beyond the Arctic Circle [29,31] (Figure 1). The distribution of its close relative, the northern red-backed vole (or red vole), *M. rutilus*, is restricted to northern parts of the circum-boreal zone, extending to the Arctic zone [29,32]. The red vole occurs from northern Fennoscandia and northern Eurasia, where it is partly sympatric with the bank vole, to the northern latitudes in North America. Introgressive hybridization between the bank vole and the red vole has been documented across a vast geographic area, from Sweden to central and southern Russia [33,34] including northern Finland [27]. All these cases showed introgression of mtDNA alone and no introgression of the nuclear genome has been reported [27,28].

In this work we revisited a geographic region where mtDNA introgression between *M. glareolus *and *M. rutilus *has been previously described [27]. First, using sequence data both from mtDNA and nuclear DNA, we confirm that mtDNA introgression from *M. rutilus *into *M. glareolus *occurs naturally in Finland, with the introgressed haplotype being fixed in the northernmost populations. Since the mitochondria house the energy machinery of the cells, we analyzed variation in two morpho-physiological traits (body size and basal metabolic rate) among *M. glareolus *populations, and found differences between females from populations with different mtDNA types, although the effect of geography could not be excluded. The underpinnings and consequences of this variation are discussed.

## Results

### Genetic variation

**Figure 2:**
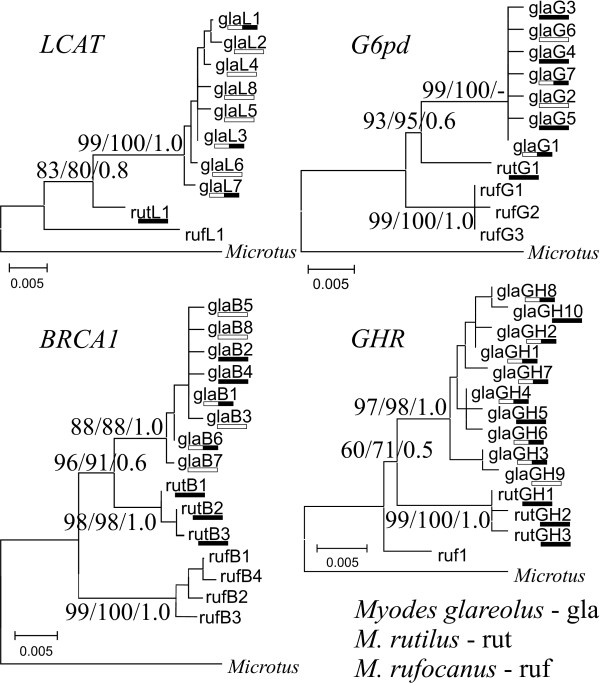
**Neighbour Joining (NJ) trees for four nuclear markers**. Numbers in the species nodes represents percentage of bootstrap values for 1000 pseudo replicates for NJ and maximum likelihood analyses and Bayesian posterior probabilities. Branch length is proportional to the number of substitutions per site. *Myodes glareolus *(gla) and *M. rutilus *(rut) haplotype names are underlined with horizontal bars referring to the type of the mtDNA detected in the particular haplotype (white, black, and black and white: GLA, RUT and both mtDNA types, respectively). Trees were rooted with sequences of *Microtus *species [GenBank: GQ267517, AB086024, AY295009, AM910792]. "-" refer to unresolved node by specific method.

**Figure 3:**
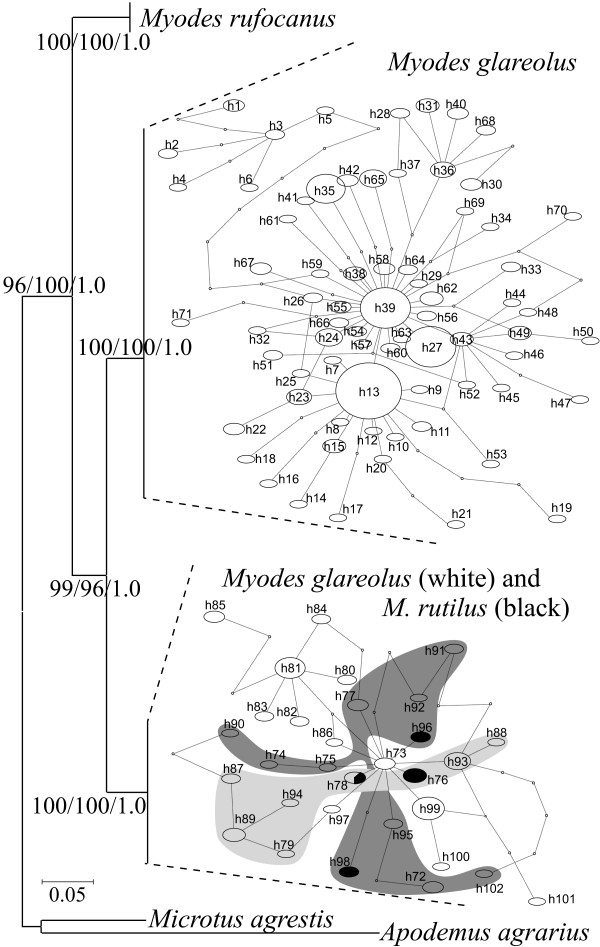
**Neighbour Joining (NJ) trees for cytochrome b**. Numbers in the species nodes represents percentage of bootstrap values for 1000 pseudo replicates for NJ and maximum likelihood analyses and Bayesian posterior probabilities. For simplicity the tree is collapsed into the major clades. Branch length is proportional to the number of substitutions per site. Haplotype networks for mtDNA of native (GLA) and introgressed *Myodes glareolus *(RUT) together with *M. rutilus *are presented separately. Oval sizes are proportional to the number of sampled individuals. Points on the branches indicate hypothetical haplotypes. Shadings for the introgressed haplotypes refer to populations: light gray - 4, Sotkamo (CE), dark gray - 6, Savukoski (NE), no shading - 5, Kolari (NW). Trees were rooted with sequences downloaded from GenBank (*Microtus agrestis *and *Apodemus agrarius*, AY167187 and AB303226).

In total, 201 voles trapped during summer 2008 in 6 localities in Finland (Figure 1) were analyzed for variation in mitochondrial and nuclear markers (Additional file 1: Table S1, Additional file 2: Table S2, Additional file 3: Table S3, Additional file 4: Table S4 and Additional File 5; Accession numbers: [GenBank: JF929975-JF930131]). After allelic phase determination (number of removed haplotypes per gene, with a lower probability of 0.95, for *LCAT*, *G6pd*, *BRCA1 *and *GHR *were 1, 0, 0 and 5, respectively) the number of alleles present in *M. glareolus *nuclear genes varied between 7 and 10 (Table 1). The phylogenies reconstructed for each of the nuclear genes were consistent across methods and coincided with the expected assignment of individuals to the species (Figure 2), since both *M. glareolus *and *M. rutilus *(see geographic distribution in Figure 1) were recovered as monophyletic with high statistical support (Figure 2). The *cyt b *sequences obtained were most likely of mitochondrial origin and not of nuclear integrated copies, as the reading frame was unbroken (no stop codons were found) and the composition of the third codon position was typical (A 40.5%, C 39.4%, G 2.2% and T 17.8%) compared to the average in mammals (A 39%, C 36%, G 3% and T 21%; [35]). Polymorphism was found in 124 sites (137 when including Swedish population and individuals trapped in 2009 in Central East Finland) which defined 81 haplotypes (99 when including Swedish and 2009 individuals) within *M. glareolus *(Table 2). Contrary to inferences based on the sequences of the four nuclear genes, the *cyt b *haplotypes sampled in *M. glareolus *did not form a monophyletic clade, and was instead split into two clades, only partially overlapping with species assignment. One clade, the *M. rutilus-*type mtDNA (RUT), grouped 57 (65 when including Swedish and specimens sampled 2009) individuals of *M. glareolus *together with *M. rutilus *specimens, suggesting mtDNA introgression. The second clade, *M. glareolus*-type mtDNA (GLA), only grouped haplotypes specific for *M. glareolus *(Figure 3). The geographic distribution of each of these haplogroups was partly disjoint, with Southern and Central Finland (1-4 on Figure 1) being occupied by the GLA type mtDNA, whereas the North and Central-East (4-6) populations had the RUT type. One population located in Central-Eastern Finland exhibited both mitotypes (population 4: Figure 1).

**Table 1:** Sequence diversity and neutrality tests for nuclear markers of bank voles.

Gene	*n_i_*	*n_h_*	*n_p_*	*h*	*π *(%)	*Θ_(S) _*per site (%)	Tajima's *D*	Fu's *F_s_*
LCAT	386	8	6	.51(.02)	.15(.12)	.16(.07)	-.15	-1.43
G6pd	388	7	6	.08(.02)	.01(.03)	.16(.07)	-1.72^&^	-11.66*
BRCA1	388	8	7	.48(.02)	.09(.08)	.19(.08)	-1.05	-3.62
GHR	376	10	11	.74(.02)	.36(.22)	.27(.10)	.72	2.23

*n_i_*, number of sampled chromosomes; *n_h_*, number of haplotypes; *n_p_*, number of polymorphic sites; *h*, haplotype diversity; *π*, nucleotide diversity; *Θ_(S)_*, computed from the number of segregating sites. Standard deviations are shown in brackets. ^&^and * indicate *p *< 0.05 and < 0.001, respectively.

**Table 2:** Sequence diversity and neutrality tests for *cyt b *of bank voles with different mtDNA types (GLA, RUT) and from different populations (see map: Figure 1).

	*n_i_*	*n_h_*	*n_p_*	*h*	*π *(%)	*Θ_(S) _*per site (%)	Tajima's *D *	Fu's *F_s_*
All data	201	81	124	.97(.01)	3.28(1.59)	2.11(.51)	1.50	-6.13
GLA type mtDNA	144	57	65	.95(.01)	.28(.17)	1.23(.31)	-2.39*	-26.64*
RUT type mtDNA	57	24	29	.95(.01)	.37(.21)	.66(.21)	-1.42^+^	-12.51*
*population/mtDNA type*								
Sweden/GLA	10	6	9	.89(.07)	.31(.20)	.33(.17)	-.37	-.88
SW/GLA	34	14	20	.87(.04)	.19(.13)	.51(.19)	-2.12*	-8.34^&^
SE/GLA	35	8	9	.75(.06)	.14(.10)	.23(.10)	-1.15	-2.42
CW/GLA	43	23	27	.96(.01)	.31(.19)	.65(.22)	-1.76*	-16.49^&^
CE/GLA, RUT	102	30	94	.94(.01)	2.32(1.14)	1.90(.49)	.73	3.94
CE/GLA	85	23	28	.92(.02)	.29(.17)	.59(.18)	-1.54*	-11.02^&^
CE/RUT	17	7	7	.83(.06)	.26(.17)	.22(.11)	.72	-.90
NW/RUT	33	12	14	.89(.03)	.28(.17)	.36(.14)	-.70	-3.37^+^
NE/RUT	15	9	17	.92(.04)	.44(.26)	.55(.23)	-.77	-1.63

*n_i_*, number of individuals; *n_h_*, number of mtDNA haplotypes; *n_p_*, number of polymorphic sites; *h*, haplotypes diversity; *π*, nucleotide diversity; *Θ_(S)_*, computed from the number of segregating sites. Standard deviations are shown in brackets. ^+^, ^&^and * indicate *p *< 0.055, < 0.05 and < 0.001, respectively.

**Figure 4:**
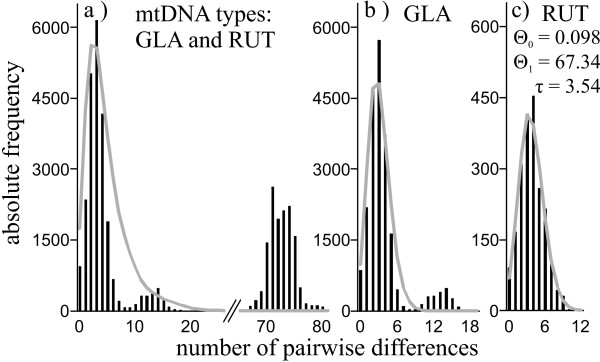
**Observed (black bars) and expected (gray lines) mismatch distributions**. Figures include: a) all samples of bank voles, *Myodes glareolus*, b) bank voles with native mtDNA type (GLA) and c) bank voles with mtDNA type of red voles *M. rutilus *(RUT). Values of the expansion parameters are only shown if the assumptions of the Sudden Expansion Model are fulfilled (unimodal distribution and goodness of fit test: p > 0.05).

Nuclear DNA sequence variation within *M. glareolus *was also detected (Table 1). The frequency spectrum of mutations did not significantly differ from the neutral mutation-drift expectations for most genes, as determined by Tajima's D and Fu's Fs (Table 1). For *cyt b*, sequence diversity of individuals with the GLA and RUT mitotype was comparable and high (Table 2). GLA and RUT type *cyt b *sequences differed by 0.098 (0.014) and 0.102 (0.014) substitutions per site, respectively, from *M. rufocanus *sequences, while the divergence between GLA and RUT mitotypes was 0.070 (0.011) (net between group average distances and its standard errors calculated by bootstrapping 1000 times). The mismatch analysis of *cyt b *sequences from *M. glareolus *showed a bimodal distribution of the number of pairwise differences (Figure 4a), which illustrates the existence of two divergent lineages. Similar results were obtained for the population from East-Central Finland (population 4, Sotkamo; Additional file 6: Figure S1d), where the mtDNA lineages coexist. The mismatch distributions analyzed separately for each of the mtDNA type (Figures 4b and 4c) and population were unimodal (Additional file 6: Figure S1). The goodness of fit test for deviation from the expectation under the Sudden Expansion Model rejected the model for the data set including all bank voles with their own mtDNA (Figure 3b; p = 0.019). All significant Tajima's *D *and Fu's *F_s _*values were negative (Table 2). Both the unimodality of the *cyt b *mismatch distribution within clades/populations and negative and significant values of tests of selective neutrality are expected for populations undergoing recent demographic growth or/and under positive selection. Given the average rate of cytochrome b divergence for rodents [36] of 0.176% per site per Myr and the parameters derived from the mismatch distribution (*τ *= 3.5; CI = 1.9-4.7) for the introgressed haplogroup, this translates to an expansion time of approximately10 500 (5 500-14 000) years ago. The unimodal distribution and star-like network may imply that introgression of mtDNA happened only once into the Finnish populations of bank voles (Figure 3). Despite the excess of rare alleles that were generally detected in *M. glareolus *in the *cyt b *and not in the nuclear genes, the multilocus HKA test [37] did not reject the null neutral model of evolution (p > 0.05).

### Phenotypic variation

**Figure 5:**
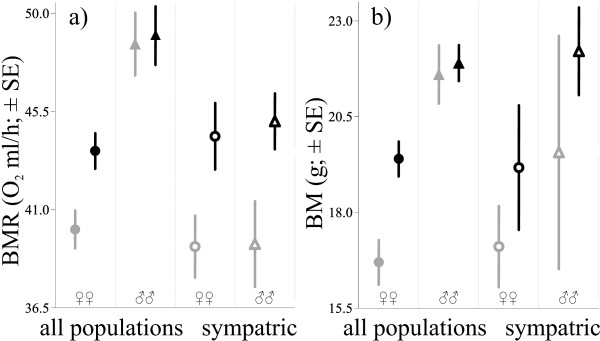
**Differences in phenotype between mitotypes**. Differences in means (± SE) of a) basal metabolism (BMR) and b) body mass (BM), between native (black) and introgressed (gray) mtDNA types in *Myodes glareolus*. Data are presented separately for females (circles) and males (triangles) captured in allopatric (filled) or sympatric populations (open figures).

All traits measured were positively correlated (N = 191, r = 0.66, 0.57 and 0.43 for BMR-BM, HW-BM and BMR-HW; p < 0.001) and all repeatability estimates (coefficients of intraclass correlation) were high and statistically significant (τ > 0.62, p < 0.0003, Table 3). As body mass and BMR were significantly influenced by a latitude-sex interaction in the initial analyses of variance (logBM: F = 3.13, p = 0.046, logBMR: F = 3.41, p = 0.035, Additional file 7: Figure S2), further tests were performed separately within sexes. Whole body BMR was higher in the GLA than in the RUT mitotype in *M. glareolus *females (p = 0.032; Table 4, Figure 5a). For males, whole body and mass-corrected BMR decreased toward the North (p = 0.002 and 0.015) but did not differ between mitotypes (the effect was generated mainly by Southern populations, which had the highest values of BMR, Additional file 7: Figure S2). Female *M. glareolus *with the GLA mitotype were significantly heavier than females with the RUT mitotype (p = 0.004, Figure 5b). Longitude, latitude and their interaction did not affect variation in females' body mass and were sequentially excluded from the model (p > 0.1). Within the sympatric population of two mitotypes (Figure 1; population 4), 8 females and 8 males of *M. glareolus *were detected with *M. rutilus *mtDNA (Table 4). The introgressed females had significantly lower values of whole-body BMR than sympatric females with native mtDNA (p = 0.049, Figure 5a). Mass corrected BMR showed the same trend but was not significant (p = 0.262). These effects were also similar for males and in the same direction, but were also insignificant. No significant differences between sympatric mitotypes in body mass were detected in this population (Table 4; Figure 4b).

**Table 3:** Repeatabilities of body mass, head width and basal metabolic rate.

	ANOVA			ANCOVA (with BM)		
Trait	N	τ	p	N	τ	p
logBM	26	.87	< .0001	26		
logHW	25	.83	< .0001	25	.74	< .0001
logBMR	26	.80	< .0001	26	.62	< .0003

τ, coefficient of intraclass correlation based on two repeated measurements across in average 35.5 days (min 16, max 51), based on variance components from ANOVA with study individual as categorical predictors, or from ANCOVA with body mass included as covariate (which provides repeatability of mass-independent trait values).

**Table 4:** Phenotypic differences between bank voles with different mtDNA types.

	All populations						Sympatric population				
	
	mitochondrial DNA type of						mitochondrial DNA type of				
	*Myodes rutilus *		*M. glareolus *		mtDNA	**lat**.	*Myodes rutilus *		*M. glareolus *		mtDNA
	
Traits	N	mean(SD)	N	mean(SD)	p	p	N	mean(SD)	N	mean	p
*Females*											
BM	20	16.7(2.72)	63	19.4(3.84)	.004*	-	8	16.8(2.77)	29	19.7(4.56)	.098
HW		13.2(.370)		13.3(.370)	.163	-		13.1(.395)		13.2(.426)	.317
BMR_ANOVA_		40.1(4.12)		43.7(6.80)	.032	-		39.3(4.16)		44.4(8.47)	.049
BMR_ANCOVA_		42.6(4.86)		42.9(4.73)	-	.445		-.351(1.17)		.097(.928)	.262
*Males*											
BM	37	21.6(4.79)	71	21.9(4.23)	-	.135	8	19.8(4.41)	47	21.4(4.35)	.329
HW		13.4(.450)		13.4(.420)	-	.265		13.2(.522)		13.4(.411)	.189
BMR_ANOVA_		48.6(8.99)		49.0(11.67)	-	.002*		39.4(5.68)		45.1(9.12)	.374
BMR_ANCOVA_		48.9(8.80)		48.9(8.83)	-	.014*		-.124(.761)		.021(1.03)	.705

Variation in body mass (BM, g), head width (HW, mm) and basal metabolic rate (BMR, ml O2 min-1) is presented for bank voles from "all populations" and from Sotkamo "Sympatric population" where mitotypes coexist. Significance of the effects: type of the mtDNA (rutilus vs. glareolus), latitude (lat.) and longitude (insignificant and excluded from table) of population (6 populations) were tested in ANOVA or ANCOVA (for BMR with BM as covariate) models on log transformed traits as dependent variables. Marginal means (and their SDs) for mtDNA types for ANCOVA models were calculated accounting for variation in BM. The insignificant effects (p > 0.1) were hierarchically reduced from the analyses of variances "-" and are not presented in the table. Latitude×longitude interactions affected only males log transformed and residual values of HW (p < 0.045), not included in the table.

* effects significant after implemented Bonferroni correction.

## Discussion

Although mitochondria serve important physiological functions in organisms, its evolution has traditionally been considered neutral. Accordingly, mtDNA variation should have little phenotypic consequences in natural populations [26]. However, during the last decade evidence is accumulating that the evolution of mtDNA is often subject to natural selection [38,39]. Over 20 years ago, mtDNA flow between two species of *Myodes *voles, the bank vole, *Myodes glareolus*, and the red vole, *M. rutilus*, was first described [27], providing a potentially good model to test whether naturally occurring mtDNA introgression may influence phenotypic traits. Here, in addition to sampling some *M. rutilus *specimens, we sampled *M. glareolus *in 6 Finnish populations, including the northern range of the species where it overlaps with *M. rutilus*, where mtDNA introgression might occur. We assessed mtDNA introgression by comparing the mtDNA-based phylogeny with that of 4 nuclear DNA markers. As expected, we detected congruence between species assignment of the individual voles with the variation in four nuclear markers (Figure 2), and found that most of the mtDNA haplotypes sampled in *M. glareolus *from northern Finland (Figure 1) cluster within the clade of *M. rutilus *(Figure 3), which is a typical consequence of mtDNA introgression [18-21,23]. Indeed, it is striking to note that the mtDNA haplotypes from *M. rutilus *are fixed in the northern populations of *M. glareolus *and only in one population did we find both the native and the introgressed mitotypes (Figure 1).

Having identified among the sampled specimens those with native and introgressed mtDNA, we aimed to test whether introgression explains differences in two key phenotypic measures: body size and basal metabolic rate. These traits were studied because size and energetic physiology are important predictors for life history traits in animals [8,12,40]. Also, some of the most recent data and theoretical predictions link variation in those traits with different fitness components [1,2,6]. We found that the repeatability of size and metabolism was significant (Table 3) and that phenotypes varied markedly between distantly located populations of bank voles. It has been postulated that the geographic variation in morpho-physiological traits might be caused by climatic differences among distant localities [10,41]. If so, the phenotypic differences are predicted to be noticeable and caused by phenotypic plasticity or adaptation to different habitats along environmental gradients, which here could include information inherited in mtDNA as a possible mechanism of physiological adaptation [42].

An interesting observation from our results is that the northern (and introgressed) *Myodes glareolus *voles were smaller and had lower BMR values than voles from the southern populations (and with the native mitotype, Additional file 7: Figure S2). Sex specific tests showed that females with different mtDNA types differed in body mass and absolute (whole-body) metabolic rate but not in mass-corrected BMR, whereas males from northern populations had lower values of absolute and mass-corrected BMR, but not body mass, and their traits did not vary significantly between mtDNA types (Table 4). Whether the inferred differences across populations are due to mtDNA introgression or related to genome reticulation is difficult to assess because bank voles from northern populations are also exclusively introgressed. To circumvent this problem we analyzed a subsample of the data from the single population where both mitotypes coexist, thus eliminating the geographic factor. Although we did not find a significant difference in body size between introgressed and non-introgressed voles (Figure 5b), we found that introgressed females had lower absolute BMR values than non-introgressed ones (Table 4, Figure 5a). However, this association was not significant when BMR was mass-corrected. Though, only 8 females with *M. rutilus *mtDNA type were sampled in this population, which yields little power to detect effects on body size and mass corrected BMR. The absence of a strong relationship between mtDNA type and variation in phenotypic characters could have resulted from relatively recent divergence between vole species, and consequently, the functional similarity between mitochondrial and nuclear genes, as shown in recent experimental transitions of whole genomes in *Drosophila *species [43]. Otherwise, the co-introgression of some nuclear genes that have not been included in current analyses, but may be important for energetic physiology and body growth rate, may have balanced the incompatibility and influenced particular phenotypes [44,45]. The observed sex-specific effects of mtDNA on phenotypes, visible at least in the between-population analyses carried out here and in another study [46], could be explained by different requirements between sexes connected to their reproductive biology [8,47]. In fact, recent models predict e.g. that the level of the rate of basal metabolism in endotherms is a consequence of directional selection operating on reproductive performance [48,49]. Accordingly, such processes are related to two energetically-costly female reproductive processes: pregnancy and lactation. It has been also experimentally confirmed that directional selection can perform differentially on size and metabolic rate between sexes in mammals [1,2]. However, the most important prediction for differential effects of mtDNA on phenotype comes from the fact that in mammals, mitochondria are almost exclusively transmitted through females. Consequently, mtDNA can only have direct adaptive effect for females and the mtDNA effects on male phenotype are expected to result from counterbalancing effects of nuclear genes [50].

The prevalence of the introgressed mtDNA haplotype in the northern populations of *M. glareolus *in extreme frequencies - the foreign haplotype seems fixed in these populations - is striking, and we thus raised the hypothesis that mtDNA introgression could have been promoted by natural selection. We assessed if the frequency spectrum of mutations of the introgressed haplotypes could be biased towards an excess of rare alleles, a pattern compatible with post-introgression positive selection. Our analysis of sequence variation among the introgressed *cyt b *haplotypes indeed shows a pattern that is not compatible with a model of population equilibrium (bell-shaped mismatch distribution and negative and significant Tajima's *D *and Fu's *F_s _*values; Table 2). This could result from adaptive introgression of mtDNA, promoting the expansion of the novel haplotype northward. However, selection is expected to affect solely the locus in question, while the effect of population expansion and other demographic phenomena should affect the whole genome equally, on average. As the HKA test did not indicate any deviation from neutral expectations of the multilocus ratios of divergence to polymorphism (see Results) and both mitotypes identified in *M. glareolus *(GLA, and the introgressed RUT) showed similar expansion signals, our results suggest that the species itself may have undergone recent population expansion, spreading the traces of mtDNA introgression throughout the northern range of *M. glareolus*. Indeed, if hybridization occurs between a resident and an invading species, introgression is expected to occur into the spatially expanding one, in a stochastically neutral phenomenon [51,52]. This scenario has been used to explain massive mtDNA introgression among hares [53,54] and (potentially) in other organisms [55]. In this demographic replacement process, markers with lower intraspecific migration rates, as is often the case with mtDNA, are expected to introgress more easily because the influx of native alleles into the invasion front is lower [51,52]. Thus, the northern expansion of *M. glareolus *into the range of *M. rutilus *could have favoured mtDNA introgression in a purely neutral manner, and could also explain the asymmetry of the observed introgression. Such asymmetry could be explained by female-biased assortative mating [56]. In a situation of interspecific contact with imbalanced frequencies of the interacting species, the females of the rare species (in this case, presumably the out-competed *M. rutilus*) tend to mate more easily with the heterospecific males, i.e., the expanding *M. glareolus*. The continuous backcrossing of hybrids with the same frequency-dependence over generations would cause introgression of the maternally transmitted mtDNA in the direction of the more abundant species (*M. glareolus*). This asymmetry could also occur if the invasion of the range of *M. rutilus *by *M. glareolus *was pioneered by males, which are known to disperse farther than females in this species [57].

## Conclusion

This study uncovered marked differences in body mass and BMR across Finnish populations of *M. glareolus *along a north-south axis. These differences correspond with massive mtDNA introgression from *M. rutilus *into the populations of *M. glareolus*, which suggests that genome reticulation may presumably be related to the differences in phenotype. Given its role in energy processing, mtDNA is an obvious candidate to underlie physiological phenotypic differences more directly. Although a signal of association between mtDNA introgression and whole-body BMR was detected from the analysis of the population where the native and foreign mtDNA haplotypes coexist, the results failed to show a relationship between mass-corrected BMR or body mass alone and the mitochondrial DNA. Further analyses using increased sample sizes and more populations where mtDNA types exist in sympatry will help to clarify this result. Also, the co-introgression of nuclear elements may be responsible for the altered phenotype and counterbalance eventual incompatibilities between nuclear and mitochondrial genomes. Obvious candidates for co-introgression with mtDNA are nuclear genes involved in physiological pathways, most notably those involved in mitochondrial function, which are known to co-evolve with mtDNA [16]. The construction of congenic strains in breeding experiments [58,59], where the mtDNA form *M. rutilus *is fixed in a nuclear background of *M. glareolus*, would allow testing if mtDNA alone alters phenotype, and ultimately, whether the mtDNA introgression has any significant fitness effects.

## Methods

### Sampling

Voles were trapped in six populations along North-South and East-West gradients in Finland, near the towns of Tammela (SW Finland: 60°48'N:23°58'E), Virolahti (SE: 60°35'N:27°34'E), Kannus (CW: 63°50'N:23°55'E), Sotkamo (CE: 64°07'N:28°23'E), Kolari (NW: 67°19'N:23°46'E) and Savukoski (NE: 67°17'N:28°09'E; Figure 1) in July and early of August 2008. Trappings were repeated during August 2009 in Sotkamo, where two mitotypes coexist in sympatry. Voles were trapped using 200 to 300 live traps in each population (Ugglan Special multiple-capture, Grahnab, Hillerstorp, Sweden). Several trap lines (≥ 9) were distantly distributed from each other (> 2 km) to lower the chance of trapping close relatives. Captured voles were assigned to species and individually marked with transponders (ID-100, Trovan). These species are easily distinguishable since *M. rutilus *has a bright red back, which is much darker in *M. glareolus*, and the relative tail to body length differs greatly between the species [0.50 in *M. glareolus *and 0.25 in *M. rutilus*; [60]]. After capture (> 30 specimens per population) the voles were transported to the laboratory where they were housed in individual cages with wood shavings and hay as bedding, in a 16L:8D photoperiod and 20 ± 2°C with standard food (Labfor 36, Lactamin AB, Stockholm, Sweden) and water available *ad libitum*. The use of the animals adhered to ethical guidelines for animal research in Finland (permission numbers: ESLH-2008-04660/Ym-23 and ESLH-2009-09663/Ym-23) as well as all the institutional guidelines.

### Molecular laboratory procedures

Genetic polymorphism in the mitochondrial cytochrome b (*cyt b*) and 4 nuclear genes (*LCAT *- lecithin-cholesterol acyltransferase exons 2 through 5, *G6pd *- Glucose-6-phosphate dehydrogenase partial intron, *BRCA1 *- Breast cancer 1 gene partial exon 11, *GHR *- growth hormone receptor partial exon 10) were analyzed for 264 voles trapped in 6 localities (including 53 voles trapped in 2009, Figure 1) in Finland and in one locality in Sweden (10 individuals). Additional reference sequences from these genes for *Apodemus, Microtus *and *Myodes *species were included [GenBank: GQ267517, AB086024, AY295009, AM910792, AY167187 and AB303226]. Total genomic DNA was extracted from ethanol preserved pieces of ear (2-3 mm^2^) collected during field expeditions using a Qiagen extraction protocol. Purification was conducted in a KingFisher apparatus. The *cyt b *gene was amplified using primers specific to the bank vole (Additional file 1: Table S1) [33]. PCR reactions for *cyt b *were performed in 30 μl volume mixes containing 3 μl of DNA, 0.24 μl of Taq polymerase, 1.8 μl of F and R primers (5 μM), 1.2 μl of MgCl2 (2 mM), 3 μl of dNTP, 3 μl of reaction buffer and 15.96 μl of H_2_O. Reactions for nuclear genes were performed on individuals trapped in 2008 in Finland as described above with following modifications including usage of 1X PCR buffer (BioTools), 200 μM of dNTPs, 0.5 U of Taq polymerase and optimum MgCl2 concentrations and specific primers (Additional file 1: Table S1). PCR reactions were performed using the following protocol: 3 min of preliminary activation of Taq polymerase at 95°C were followed by 32 (34 for nuclear genes) three-step cycles of denaturation at 94°C (30 sec), annealing temperature specific for primer (30 sec; see Additional file 1: Table S1) and extension at 72°C (70 sec), and a final extension at 72°C (10 min).

PCR products were purified by the Exo - SAP assay (Amersham Biosciences). Sequence analysis was performed with the BigDye Terminator v3.1 cycle sequencing kit (Applied Biosystems). Sequencing reactions were performed using the PCR primers (Additional file 1: Table S1) in 16 μl mixes containing 1 μl of primer (3.3 μM) and 3 μl of PCR product according to ABI sequencing protocol. Sequencing was conducted in two directions using a BigDye Terminator kit (Applied Biosystems) on an Applied Biosystems 3130xl Genetic Analyzer. Both forward and reverse strands were merged using the SeqScape v 2.1.1 and aligned using ClustalX v 2.0.10 softwares.

### Sequence analyses

Initially, the allelic phase determinations for each of the nuclear gene were obtained with PHASE v2.1.1 [61]. In the subsequent phylogenetic analyses, only alleles with phase calls with posterior probabilities over 0.95 were used. DnaSP v 5.10.01 [62] and ARLEQUIN 3.11 [63] were used to detect the number of haplotypes and number of variable sites within each gene and species. Phylogenetic relationships among sequences were reconstructed using three different methods. First, using the Neighbour Joining method, phylogenies were inferred from a distance matrix obtained with the Jukes-Cantor nucleotide substitution model and the robustness of the trees were assessed by bootstrap resampling (BS: 1000 random replications) in MEGA 4 software [64]. Based on the reconstructed initial trees a hierarchical likelihood ratio test and AIC based model selection were then conducted in ModelTest v 3.0 [65], as implemented in HyPhy package [66] which determined the simplest model of sequence evolution that best fits variation in data. The chosen models were further included in the phylogenetic reconstructions. The robustness of Maximum-Likelihood phylogenetic analyses (ML) [67] was assessed by bootstrap resampling (1000 random replications) performed in the software PHYML v 2.4.4 [68]. The Bayesian inference (BI) approach was applied to reconstruct the phylogenies using the program MrBayes v 3.1.2 [69]. Four Markov Chain Monte Carlo (MCMC) simulations were started from the tree with random topology and branch lengths. Simulations were run for 10 million (1 million for nuclear markers) generations with trees sampled every 100 generations (100 000 trees saved) with the first 7 000 (700 for nuclear markers) trees discarded from further analyses (burn-in). The remaining trees were used to construct the consensus tree and estimate posterior probabilities for all nodes in the BI tree using the 50% majority rule.

The relationships among *cyt b *haplotypes were additionally analyzed and visualized with the statistical parsimony method implemented in TCS 1.21 [70]. These analyses were run together and separately for two types of mtDNA, bank vole - GLA and red vole - RUT (the latter group included haplotypes found both on bank voles and red vole specimens). We also estimated nucleotide diversity (π), θ_s _computed from number of segregating sites, haplotype diversity (h) and mismatch distributions (ARLEQUIN 3.11) [63]. The mismatch distributions were calculated [71] and the goodness-of-fit tests of the observed to the expected distributions according to the Sudden Expansion Model were tested [72]. The Sudden Expansion Model assumes that initial population at equilibrium (of size: θ_0_) grew rapidly to a new size (θ_1_), mutational times ago: τ = 2*ut *(u - mutation rate, t - time since the expansion in generation). The confidence intervals for τ were calculated with 1000 bootstrap replicates for the alpha level of 0.010. The assumption of selective neutrality and population equilibrium were tested with Tajima's D [73] and Fu's F_s _[74] statistics with 5000 simulated samples.

Multilocus ratios of polymorphism in *M. glareolus *to divergence between this species and *M. rufocanus *were contrasted with the expectations of a neutral model using a Hudson-Kreitman-Aguade (HKA) test [37] as implemented in the HKA program (http://lifesci.rutgers.edu/~heylab/).

### Metabolic measurements

After two months of acclimatization to laboratory conditions, measurements of oxygen consumption (ml h^-1^) were conducted in an eight-channel open-flow respirometric system (Sable Systems, Henderson, NV) based on the Fc-1B O_2 _(Sable Systems) analyzer. The system was adapted to measure 7 animals (with the eighth channel as a reference) per day. Prior to measurements, animals were weighed and placed in Plexiglas chambers (180 ml) without water or food at 30.0 ± 0.5°C, which is within the species thermal neutral zone. The chambers were connected to a system of dry air flow (after passing through a column with silica gel) of approximately 260 ml min^-1 ^(accurate flows were measured with FlowBar, Sable Systems, Henderson, NV, and included in metabolic rate calculations). Oxygen consumption was recorded for a period of 7 h 30 min (from 09:00 to 16:30). The samples of dried air (passed through Drierite desiccant) were taken sequentially from the seven occupied measurement chambers and the reference chamber every 15 min (with flow rate: 150 ml min^-1^). In each cycle, each measurement chamber was active for at least 110 seconds (first and reference chambers were active 10 sec longer each) during which O_2 _values were sampled every 1 sec. From each chamber, measurements taken during the last 20 seconds were used for the calculation of average oxygen consumption. Oxygen consumption (metabolic rate) was calculated using O_2 _measures according to the formula VO_2 _= {V_i _(FdO_2_)/[1 - FeO_2 _(1 - RQ)]}, [equation 1b from [75]] where VO_2 _is the oxygen consumption rate, V_i _is the flow rate measured before measurement chamber, FdO_2 _is the difference of O_2 _fractional concentrations in dry air flow before and after passing through the measurement chamber, and FeO_2 _is the fractional concentration of O_2 _in dry air flow after passing the measurement chamber. A respiratory quotient (RQ = CO_2 _eliminated/O_2 _consumed) of 0.75 for nearly starved animals was assumed in the equation [75]. A total of 29 oxygen consumption values were calculated for each animal throughout one measurement trial: every 15 minutes over the 7 h 30 min period. Since the lowest of such values may be subject to high error rates, mainly due to the different physiological status of animals prior to the measurement, we defined BMR as a mean of the third, fourth and fifth lowest values, which proved to be quite accurate [76],.

### Statistical analyses

To normalize the data (BMR was right skewed: K-S test, p = 0.005), body mass, head width and basal metabolic rate measurements were log transformed prior to analyses. Pearson partial correlations between measured characters included sex, population of origin and mtDNA type as cofactors. Repeatabilities (τ) of traits were estimated on two consecutive measurements (collected between 16 to 51 days; mean = 35.5 days) as intraclass correlation coefficients, derived either from simple analyses of variance or analyses of covariance with body mass as a continuous predictor [77]. Geographic variation in body mass (BM, g), head width (HW, mm) and basal metabolic rate (BMR, ml O_2 _h^-1^) were tested with general linear models. In the models, log transformed traits (BM, HW and BMR) were included as dependent variables, while sex, type of mtDNA (rutilus vs. glareolus), longitude (2 levels) and latitude (3 levels) of populations were included as fixed independent factors. Analyses were also conducted on residual values of BMR, derived from linear regression of log BMR on log BM.

## Authors' contributions

ZB carried out the fieldwork, phenotypic and genetic analyses, and all statistical analyses and drafted the manuscript. EK and TM participated in fieldwork and laboratory studies as well as in designing experiments and drafting the manuscript. SB conducted PCR and sequencing as well as alignment and phase analyses of nuclear markers. PCA and JM-F participated in all analyses of molecular data and in drafting and finalizing the manuscript. All authors read and approved the final version of manuscript.

## Supplementary Material

Additional file 1 **Table S1 - Primers and PCR parameters for mitochondrial and nuclear markers**. Click here for file

Additional file 2 **table S2 - Localities, species sampled, and detected haplotypes**. Click here for file

Additional file 3 **table S3 - GenBank accession numbers for *cyt b *haplotypes**. Click here for file

Additional file 4 **table S4 - GenBank accession numbers for nuclear haplotypes**. Click here for file

Additional file 5 **Sequence alignments included in the study**. Click here for file

Additional file 6 **figure S1 - Observed (bars) and expected (gray lines) mismatch distributions of Finnish populations of bank voles**. a), b) and c) refer to populations of bank voles with native mtDNA type (GLA); d), e) and f) Sotkamo population with both mtDNA types and g) and h) populations of bank voles with red voles mtDNA type (RUT). See Figure 1 for information about localizations of populations. Values of the expansion parameters are only shown if the assumptions of the Sudden Expansion Model are fulfilled. Click here for file

Additional file 7 **figure S2 - Differences in phenotype between populations**. Differences in means (± SE) of body mass (g) and basal metabolism (BMR; mL O_2 _h^-1^) between Finnish populations of *Myodes glareolus*. Data are presented separately for females (circles) and males (triangles) captured along West (filled) and East (open figures) latitudinal gradients. Click here for file
