# Supplementary material for: Introgression of mitochondrial DNA among Myodes voles: consequences for energetics?
Source: BMC Evol Biol. 2011 Dec 9;11:355. doi: 10.1186/1471-2148-11-355 (PMC3260118; doi:10.1186/1471-2148-11-355)
Supplement: Additional file 1 — Table S1 - Primers and PCR parameters for mitochondrial and nuclear markers. [file 1471-2148-11-355-S1.PDF]

Additional table S1 – Primers and PCR parameters of the genes included in the study

Gene names	Primers F/R: 5´- 3´	Ta	[MgCL] µl	Chr	Nuc
LCAT	CACCATCTTCCTGGATCTCAA	58	2	8	563
	AAGAAATACAGCACATGTAGGCA				
G6pd	CAGATCTGTGAACGTGTTTGG	58	2.5	X	577
	GGTACAACTCTTCCCTCAGG				
BRCA1	AAGACAGAATGTAGAAAAGGCTG	50.7	2.5	11	576
	ACCGACAGGCTCAAGTGCGA				
GHR	GGCGTTCATGACAACTACAAACCTGA	62	1.5	15	617
	ATAGCCACACGAGGAGAGGAACT				
cyt b	ATCATCAACCACKCATTTATT	46.1	1.2	mtDNA	954
	ATTATGCCTGCTATTGGTATG				
