# Supplementary material for: Introgression of mitochondrial DNA among Myodes voles: consequences for energetics?
Source: BMC Evol Biol. 2011 Dec 9;11:355. doi: 10.1186/1471-2148-11-355 (PMC3260118; doi:10.1186/1471-2148-11-355)
Supplement: Additional file 2 — table S2 - Localities, species sampled, and detected haplotypes. [file 1471-2148-11-355-S2.PDF]

Additional table S2 – Localities, species sampled, and detected haplotypes

Myodes	MtDNA type	Area	Locality	Cyt b haplotypes	LCAT haplotypes
glareolus	GLA	Lund, Sweden	R	h1-6	
		Tammela	1, SW	h7-8, h11-16, h18-19, h23, h25, h53, h55	glaL1, glaL3, glaL7
		Virolahti	2, SE	h9-10, h13, h17, h22, h39, h62, h66	glaL1, glaL3, glaL6, glaL7
		Kannus	3, CW	h20-21, h39, h43-52, h54, h56, h59-60, h63, h65 ,h67, h69-71	glaL1, glaL2, glaL3, glaL7
		Sotkamo	4, CE	h24, h26-42, h57-58, h61, h64, h68	glaL1, glaL3, glaL6, glaL7, glaL8
	RUT	Sotkamo	4, CE	h78-79, h87-89, h93-94	glaL1, glaL3, glaL6, glaL7, glaL8
		Kolari	5, NW	h73, h80-86, h97, h99-101	glaL1, glaL3, glaL7
		Savukoski	6, NE	h72, h74-75, h77, h90-92, h95, h102	glaL1, glaL3, glaL7
rutilus	RUT	Sotkamo	4, CE	h76, h78	
		Savukoski	6, NE	h96, h98	rutL1
rufocanus	RUF	Savukoski	6, NE	h103-106	rufL1
