# Supplementary material for: Introgression of mitochondrial DNA among Myodes voles: consequences for energetics?
Source: BMC Evol Biol. 2011 Dec 9;11:355. doi: 10.1186/1471-2148-11-355 (PMC3260118; doi:10.1186/1471-2148-11-355)
Supplement: Additional file 3 — table S3 - GenBank accession numbers for cyt b haplotypes. [file 1471-2148-11-355-S3.PDF]

Additional table S3 – GenBank accession numbers for cyt b haplotypes

hap	GenBank	hap	GenBank	hap	GenBank	hap	GenBank
h1	JF929975	h28	JF930002	h55	JF930029	h81	JF930054
h2	JF929976	h29	JF930003	h56	JF930030	h82	JF930055
h3	JF929977	h30	JF930004	h57	JF930031	h83	JF930056
h4	JF929978	h31	JF930005	h58	JF930032	h84	JF930057
h5	JF929979	h32	JF930006	h59	JF930033	h85	JF930058
h6	JF929980	h33	JF930007	h60	JF930034	h86	JF930059
h7	JF929981	h34	JF930008	h61	JF930035	h87	JF930060
h8	JF929982	h35	JF930009	h62	JF930036	h88	JF930061
h9	JF929983	h36	JF930010	h63	JF930037	h89	JF930062
h10	JF929984	h37	JF930011	h64	JF930038	h90	JF930063
h11	JF929985	h38	JF930012	h65	JF930039	h91	JF930064
h12	JF929986	h39	JF930013	h66	JF930040	h92	JF930065
h13	JF929987	h40	JF930014	h67	JF930041	h93	JF930066
h14	JF929988	h41	JF930015	h68	JF930042	h94	JF930067
h15	JF929989	h42	JF930016	h69	JF930043	h95	JF930068
h16	JF929990	h43	JF930017	h70	JF930044	h96	JF930076
h17	JF929991	h44	JF930018	h71	JF930045	h97	JF930069
h18	JF929992	h45	JF930019	h72	JF930046	h98	JF930077
h19	JF929993	h46	JF930020	h73	JF930047	h99	JF930070
h20	JF929994	h47	JF930021	h74	JF930048	h100	JF930071
h21	JF929995	h48	JF930022	h75	JF930049	h101	JF930072
h22	JF929996	h49	JF930023	h76	JF930074	h102	JF930073
h23	JF929997	h50	JF930024	h77	JF930050	h103	JF930078
h24	JF929998	h51	JF930025	h78	JF930051	h104	JF930079
h25	JF929999	h52	JF930026	h78	JF930075	h105	JF930080
h26	JF930000	h53	JF930027	h79	JF930052	h106	JF930081
h27	JF930001	h54	JF930028	h80	JF930053		
