# Supplementary material for: Introgression of mitochondrial DNA among Myodes voles: consequences for energetics?
Source: BMC Evol Biol. 2011 Dec 9;11:355. doi: 10.1186/1471-2148-11-355 (PMC3260118; doi:10.1186/1471-2148-11-355)
Supplement: Additional file 4 — table S4 - GenBank accession numbers for nuclear haplotypes. [file 1471-2148-11-355-S4.PDF]

Additional table S4 – GenBank accession numbers for nuclear haplotypes

LCAT		G6pd		BRCA1		GHR	
hap	GenBank	hap	GenBank	hap	GenBank	hap	GenBank
glaL1	JF930082	glaG1	JF930092	glaB1	JF930103	glaGH1	JF930118
glaL2	JF930083	glaG2	JF930093	glaB2	JF930104	glaGH2	JF930119
glaL3	JF930084	glaG3	JF930094	glaB3	JF930105	glaGH3	JF930120
glaL4	JF930085	glaG4	JF930095	glaB4	JF930106	glaGH4	JF930121
glaL5	JF930086	glaG5	JF930096	glaB5	JF930107	glaGH5	JF930122
glaL6	JF930087	glaG6	JF930097	glaB6	JF930108	glaGH6	JF930123
glaL7	JF930088	glaG7	JF930098	glaB7	JF930109	glaGH7	JF930124
glaL8	JF930089	rutG1	JF930099	glaB8	JF930110	glaGH8	JF930125
rutL1	JF930090	rufG1	JF930100	rutB1	JF930111	glaGH9	JF930126
rufL1	JF930091	rufG2	JF930101	rutB2	JF930112	glaGH10	JF930127
		rutG3	JF930102	rutB3	JF930113	rutGH1	JF930128
				rufB1	JF930114	rutGH2	JF930129
				rufB2	JF930115	rutGH3	JF930130
				rufB3	JF930116	rufGH1	JF930131
				rufB4	JF930117		
