# Supplementary material for: Introgression of mitochondrial DNA among Myodes voles: consequences for energetics?
Source: BMC Evol Biol. 2011 Dec 9;11:355. doi: 10.1186/1471-2148-11-355 (PMC3260118; doi:10.1186/1471-2148-11-355)
Supplement: Additional file 6 — figure S1 - Observed (bars) and expected (gray lines) mismatch distributions of Finnish populations of bank voles. a), b) and c) refer to populations of bank voles with native mtDNA type (GLA); d), e) and f) Sotkamo population with both mtDNA types and g) and h) populations of bank voles with red voles mtDNA type (RUT). See Figure 1 for information about localizations of populations. Values of the expansion parameters are only shown if the assumptions of the Sudden Expansion Model are fulfilled. [file 1471-2148-11-355-S6.PDF]

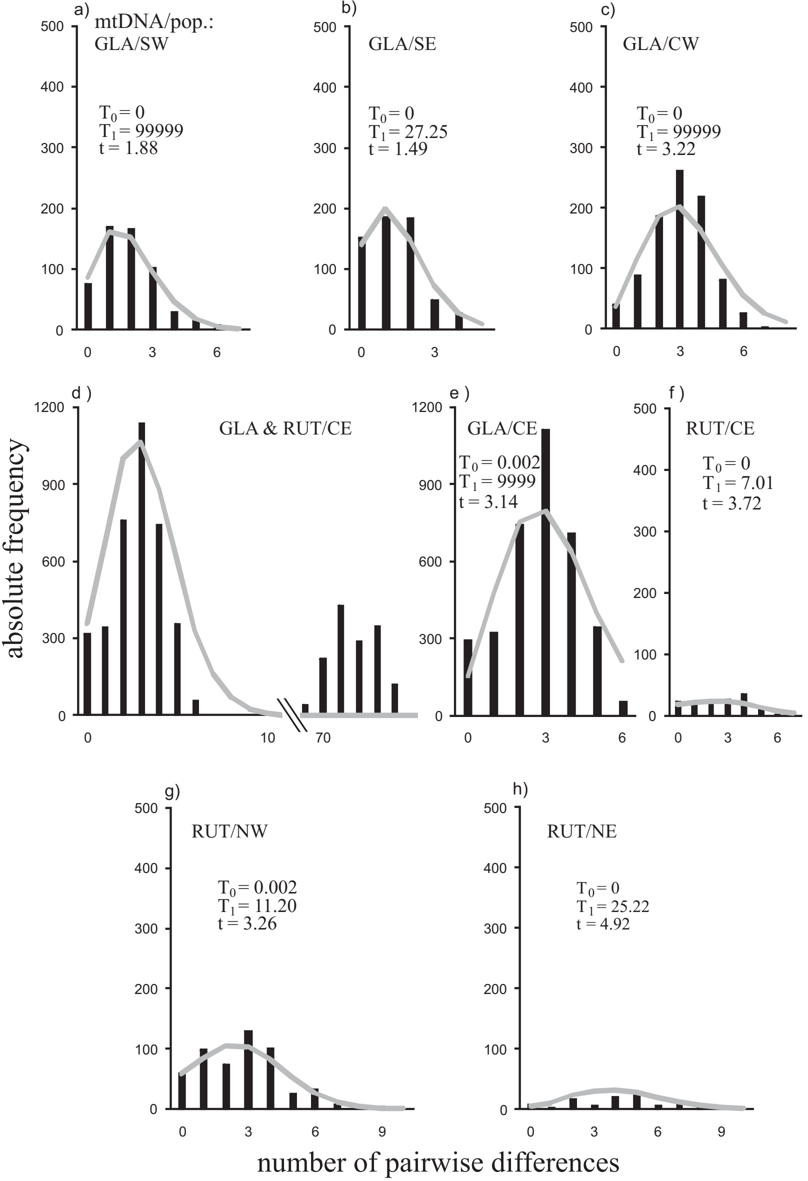

Figure S1. Observed (bars) and expected (gray lines) mismatch distributions of Finnish populations of bank voles *Myodes glareolus*. a), b) and c) populations of bank voles with native mtDNA type (GLA); d), e) and f) Sotkamo population with both mtDNA types and g) and h) populations of bank voles with red voles mtDNA type (RUT). See Fig. 1 for information about localizations of populations. Values of the expansion parameters are only shown if the assumptions of the Sudden Expansion Model are fulfilled.
