# Supplementary material for: Introgression of mitochondrial DNA among Myodes voles: consequences for energetics?
Source: BMC Evol Biol. 2011 Dec 9;11:355. doi: 10.1186/1471-2148-11-355 (PMC3260118; doi:10.1186/1471-2148-11-355)
Supplement: Additional file 7 — figure S2 - Differences in phenotype between populations. Differences in means (± SE) of body mass (g) and basal metabolism (BMR; mL O2 h-1) between Finnish populations of Myodes glareolus. Data are presented separately for females (circles) and males (triangles) captured along West (filled) and East (open figures) latitudinal gradients. [file 1471-2148-11-355-S7.PDF]

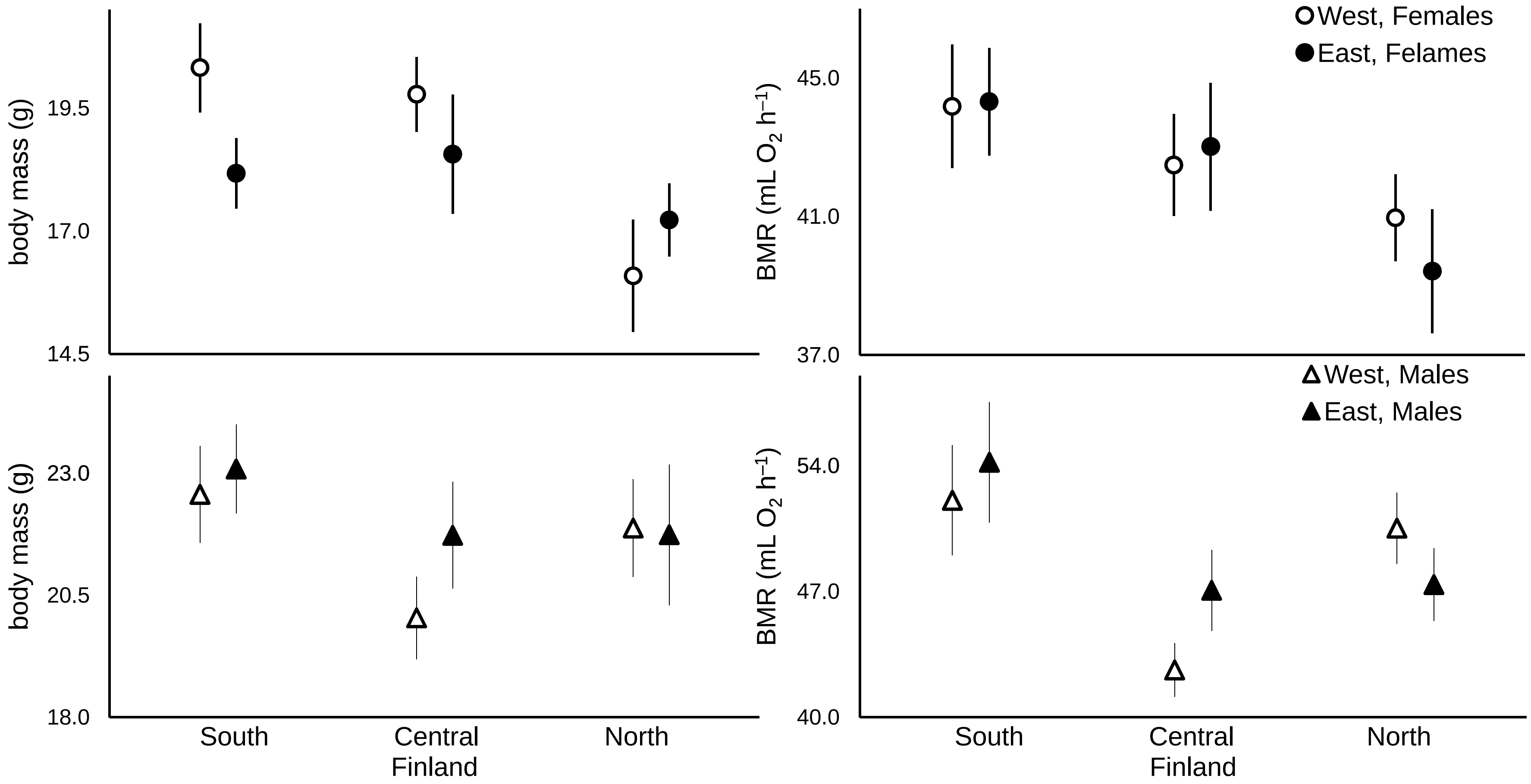

Figure S2. Differences in phenotype between populations. Differences in means (±SE) of body mass (g) and basal metabolism (BMR; mL O_2 h^{-1}) between Finnish populations of *Myodes glareolus*. Data are presented separately for females (circles) and males (triangles) captured along East (filled) and West open figures) latitudinal gradients.
